# Seven Letters with Answers

**Published:** 1871-01

**Authors:** 


### Our Correspondence.

### SEVEN LETTERS WITH ANSWERS.

PAINTED POST, STEUBEN COUNTY.
November 8th, 1870.

DR. T. S. UP DE GRAFF—

*Dear Sir*:—I am fourteen years old and the oldest of my mother's family of five children. She does her work alone, as I can give her but little assistance while attending school. I am anxious to help her all I can, and will try *hard* to get 100 subscribers to the BISTOURY, for which you kindly offer as reward, a McLean & Hooper Sewing Machine, one of which would help mother more in a week than I could with my hands, in a month. Please send me a sample copy, that I may begin the work soon, and oblige, Yours Gratefully,

BARBARA N.

—Barbara, that is a very prettily-written and well composed letter, for a girl of fourteen to write. BISTOURY receives very many letters from grown people, who do not write half so well. We have forwarded the sample copy and hope you may be successful, as we are sure you will be, in earning a sewing machine for your Mother.

PORT JERVIS, Nov. 18th, 1870.

DR. UP DE GRAFF—

*Dear Sir*:—I saw mamma's BISTOURY to-day, and read in the back part of it, that you would send one of those large Webster Dictionaries to any one who would send you fifty subscribers to your magazine. O, I'm so glad, because I have been saving my money for ever so long, to buy papa one of those very dictionaries for a Christmas present, for I heard him say he would like one. And now I send the twenty-five dollars and the fifty names that mamma has written down plainly for you, and I want you to send the book to my Uncle Ben. to keep for me, so papa won't find it out. Mamma says I must tell you how old I am. I will be nine years old the 10th of next month. It is such fun to get subscribers—I am going to send you a club of a hundred and get mamma a sewing machine.

So good bye—don't forget to send the book to Uncle———

LILLY A. B.———

—Well there, Lilly, that is what we call a real cute, little letter, from what must be a very bright little girl. We are sure your papa will be very happy when you surprise him with the beautiful and valuable book which you have earned for him.

We have ordered the publishers to send the work to your "Uncle Ben." "so that papa may not find it out."

---

AUGUSTA, GA., Oct. 30th, 1870.

DR. UP DE GRAFF—

*Dear Sir*:—I ran across your Magazine, the BISTOURY, at the house of a friend, to-day, and for the first time in my life, saw an exposition of the manner in which those fellows who advertise to furnish advice for the cure of disease *free*, make it pay. I have often wondered whether there really *could* be men so charitable as to advertise extensively, at what must be great cost to them, that they would disclose the means of curing all manner of disease, and that too without one cent of reward. It did not seem natural to have men do so, and I have often wondered where the *catch* was. Your BISTOURY explains it fully, and I am glad there is such a journal published. Wish I might have seen it long ago, I could have saved myself, and several young friends as well, much money that has been thrown away upon these benevolent clergymen (?) and others of their stamp. There is a fellow by the name of, or who calls himself, Joseph T. Inman of Station D. Bible House, New York City, who advertises very extensively that he discovered, while a clergyman in South America, as Missionary, a safe and simple remedy for cure of "Nervous Weakness," "Early Decay," &c., &c., which, "prompted by a desire to benefit the afflicted and unfortunate," he will send to any one who needs it, *free of charge*. Myself and others have sent to this miserably, benevolent old humbug for his receipt for his "remedy," and found as the BISTOURY says, that in the receipe were one or two ingredients that no druggist or any one else, save the Reverend gentlemen, knew anything about. We therefore, of course, accepted his hint to send direct to *him* for the medicine already prepared, "*if our druggist did not have all the ingredients*." We have sent him much money for his abominable, worthless trash, without, as we ought to have known, receiving one particle of benefit.

I wish, if you have not already done so, that you would give this chap a punch with your BISTOURY and save others from being humbugged as we have been. Yours Truly.

H. A. B.

—We have repeatedly exposed the manner by which those "*free receipe*" men swindle the innocents, that we had supposed their operations were pretty generally understood. We make no comments on the above letter, as it pretty thoroughly explains itself.

---

NEW LEBANON, Ind., Nov. 6th, 1870.

DR. THAD S. UP DE GRAFF, EDITOR BISTOURY—

*Dear Sir:*—Who are Sayre & Co., of 210 Broadway, N. Y., who advertise "Wyatts Great Consumption Cure." Are they trustworthy ?

J. R. S.

—Answer:—They are miserable swindlers and humbugs. There is no more virtue in their "Consumption Cure" than in their honesty. They are after your money—let them alone. Apply to your family physician, he will give you all the advice necessary in your case.

---

WATERFORD, OHIO, Nov. 1, 1870.

EDITOR BISTOURY—

Can you tell me whether the "Howard Association," who advertise to cure private diseases are reliable or not ?

J. T. R.

—They are perfectly *reliable*. They will as certain as you send your money, cheat you out of it, and your life as well, if you take their medicine. When we say *them* and *their*, we mean *he* and *his*. For the "Association" is one man and a very ignorant one at that.

WEEDSPORT, N. Y., Nov. 21, 1870.

THAD S. UP DE GRAFF, EDITOR BISTOURY—

*Dear Sir:*—Will you please inform me whether Drs. Comb and Wilson who are traveling through here treating the people for chronic diseases, are good physicians or not?

L——.

—Of course, we'll tell you. Dr. (?) Comb is a shoemaker, and we're told by Mr. Havens of your place, that he's a very poor one, too. Dr. (?) Wilson, is a broken down showman, and formerly the "Great American Tape Worm Doctor." He's a buster—he's better at balancing whips and feathers on his nose than he is at "doctoring." They're a splendid pair. Take their physic by all means, but don't meet them after night in a dark place—and keep your hen-house locked while they are in your town.

This letter explains itself:

OGDENSBURG, TIOGA CO., PA., Nov. 2, 1870.

L. A. BABCOCK, M. D.:—

*Dear Sir:*—The Uterine Supporter I received from you as a specimen sample, I applied to a case of prolapsus, with ulceration. It performed a radical cure in less than six weeks.

Please send me another by express, C. O. D., to Roaring Branch, Lycoming Co., Pa., via. Elmira, N. Y.

Send the medium size.

Respectfully Yours,

J. E. CLEVELAND, M. D.

CHEAP PAINT.—A very economical and at the same time durable paint can be made, for covering out building, fences, &c., by taking skim milk 2 quarts, fresh slacked lime 8 oz., linseed oil 6 oz., white Burgundy pitch 2 oz., Spanish white 3 lbs. The lime must be slacked in hot water, exposed to the air, and when cool, mixed in one fourth of the milk; the oil, in which the pitch must be dissolved, to be added a little at a time; then add the balance of milk and finally the Spanish white. It is applied in the ordinary manner and this quantity is sufficient to paint over 27 square yds., two coats.

—A physician writes, asking a renewal of a note which he owes, giving as a reason therefor: "We are in a horrible crises. There is not a sick man in the district."

